# Supplementary material for: Disparities and trends of the incidence and mortality of female-specific cancers in the United States
Source: PLoS One. 2025 Oct 14;20(10):e0334128. doi: 10.1371/journal.pone.0334128 (PMC12520385; doi:10.1371/journal.pone.0334128)
Supplement: Attachment — Submitted filename: Response to Reviewers.docx [file pone.0334128.s001.docx]

Response to Reviewers

Dear Dr. Kehinde S. Okunade, Dr. Adaiah Priscillia Soibi-Harry, and Dr. Adenekan Muisi Alli,

Thank you very much for your letter dated July 17, 2025, regarding the decision of our manuscript entitled “Disparities and Trends of the Incidence and Mortality of Female-Specific Cancers in the United States” (manuscript number PONE-D-25-31996), which was submitted to your excellent journal (*Plos one*). We sincerely appreciate the time and effort invested by you and the reviewers in providing such thoughtful and constructive feedback. The suggestions made by the reviewers have been invaluable in improving the quality and clarity of our manuscript. We highly value the work that has gone into reviewing our submission and are grateful for the opportunity to revise it in line with the expert feedback provided.

We have carefully addressed all the comments and suggestions and corrected typographical and grammatical errors. Our point-by-point response to the reviewers’ comments is provided on the following pages, and the revisions in the manuscript are highlighted in red for clarity.

We thank you once again for your consideration and the continued opportunity to have our work evaluated by your esteemed journal. We look forward to your favorable decision.

Kindest regards,

Yours sincerely,

Prof. Fan Li

We would like to express our sincere thanks to the academic editor and reviewers for the constructive and positive comments. Our responses are as follows:

**Replies to Academic Editor**

1. Please ensure that your manuscript meets PLOS ONE's style requirements, including those for file naming. The PLOS ONE style templates can be found at https://journals.plos.org/plosone/s/file?id=wjVg/PLOSOne_formatting_sample_main_body.pdf and [https://journals.plos.org/plosone/s/file?id=ba62/PLOSOne_formatting_sample_title_auth ors_affiliations.pdf](https://journals.plos.org/plosone/s/file?id=ba62/PLOSOne_formatting_sample_title_auth%20ors_affiliations.pdf)

**Answer:** We sincerely apologize for the oversight. We have now meticulously revised our entire manuscript according to the PLOS ONE style templates provided. This includes adjustments to text formatting, heading levels, author affiliations, reference style, and figure/table legends. All files have been renamed in accordance with the journal's guidelines. The revised manuscript has been uploaded to the submission system.

2. We noticed you have some minor occurrence of overlapping text with the following

previous publication(s), which needs to be addressed:

-DOI: 10.14309/ajg.0000000000003198

-https://doi.org/10.1016/j.eururo.2022.11.023

In your revision ensure you cite all your sources (including your own works), and quote or rephrase any duplicated text outside the methods section. Further consideration is dependent on these concerns being addressed.

**Answer:** We thank the editor for identifying this. The overlapping text, primarily in the Materials and Methods section, has been thoroughly addressed. Specifically:

1. In the Materials and Methods (Page 5, lines96-97; Page 6, lines105-106; Page 8, lines149-151), the text overlapping with the source (now cited as reference [7], DOI: 10.14309/ajg.0000000000003198) has been converted into a proper quotation and cited appropriately to acknowledge the original source.

2. We have revised the Materials and Methods section (Page 6, lines116-121; Page 7, lines130-132; Page 8, lines147-148) to properly quote and cite the text that overlapped with the source, now referenced as [11] (DOI: 10.1016/j.eururo.2022.11.023), thus ensuring due acknowledgment.

We have carefully reviewed the entire manuscript to ensure that all duplicated text outside the Materials and Methods section has been adequately resolved either through citation, paraphrasing, or direct quotation.

3. Thank you for stating the following financial disclosure:

[This study was supported by the National Natural Science Foundation of China (grant 82202913 and 82372996) and the Natural Science Foundation of Chongqing (grant CSTB 2023 NSCQ-MSX0480).]. Please state what role the funders took in the study. If the funders had no role, please state: "The funders had no role in study design, data collection and analysis, decision to publish, or preparation of the manuscript." If this statement is not correct you must amend it as needed. Please include this amended Role of Funder statement in your cover letter; we will change the online submission form on your behalf.

**Answer:** We have amended our financial disclosure statement as instructed. The following sentence has been added to the Funding section in the manuscript and is also included in our cover letter:

"The funders had no role in study design, data collection and analysis, decision to publish, or preparation of the manuscript."

4. In the online submission form, you indicated that [The data utilized in this study can be found in the Surveillance, Epidemiology, and End Results database Public-access SEER data (https://seer.cancer.gov/), National Center for Health Statistics (NCHS), and 2021 Global Burden of Disease (GBD) study (https://vizhub.healthdata.org/gbd- results/). Analytic code and any additional information required to reanalyze the data reported in this paper can be obtained from the corresponding author upon reasonable request, following the publication of this article.]. All PLOS journals now require all data underlying the findings described in their manuscript to be freely available to other researchers, either 1. In a public repository, 2. Within the manuscript itself, or 3. Uploaded as supplementary information. This policy applies to all data except where public deposition would breach compliance with the protocol approved by your research ethics board. If your data cannot be made publicly available for ethical or legal reasons (e.g., public availability would compromise patient privacy), please explain your reasons on resubmission and your exemption request will be escalated for approval.

**Answer:** We confirm that the data underlying the findings of this study are fully available without restriction. All data were sourced from publicly available databases:

The Surveillance, Epidemiology, and End Results (SEER) database (https://seer.cancer.gov/)

The National Center for Health Statistics (NCHS)

The 2021 Global Burden of Disease (GBD) study (https://vizhub.healthdata.org/gbd-results/)

As stated in the manuscript, any additional analytic code required to reanalyze the data is available from the corresponding author upon reasonable request. This statement has been verified and remains accurate in our revised manuscript.

5. Your ethics statement should only appear in the Methods section of your manuscript. If your ethics statement is written in any section besides the Methods, please move it to the Methods section and delete it from any other section. Please ensure that your ethics statement is included in your manuscript, as the ethics statement entered into the online submission form will not be published alongside your manuscript.

**Answer:** Thank you for this clarification. We have moved the ethics statement exclusively to the Materials and Methods section (Page9, lines 166-169) of the manuscript and have deleted it from any other section to comply with journal policy.

6. Please include a separate caption for each figure in your manuscript.

**Answer:** We have reviewed all figures and confirm that each now has a separate, descriptive caption directly beneath it in the manuscript file.

7. Please review your reference list to ensure that it is complete and correct. If you have cited papers that have been retracted, please include the rationale for doing so in the manuscript text, or remove these references and replace them with relevant current references. Any changes to the reference list should be mentioned in the rebuttal letter that accompanies your revised manuscript. If you need to cite a retracted article, indicate the article’s retracted status in the References list and also include a citation and full reference for the retraction notice.

**Answer:** We have conducted a complete and thorough review of our reference list. We confirm that it is accurate and complete. No retracted papers were cited in our manuscript.

We would also like to extend our sincere gratitude to you for overseeing the review process and for providing us with the opportunity to improve our manuscript.

**Replies to Reviewer #1**

This manuscript addresses a critically important topic concerning the racial, ethnic, and geographic disparities in incidence and mortality trends of six female-specific cancers in the United States. The authors utilize robust national databases (SEER and GBD) and appropriate statistical methods (Joinpoint regression, APC/AAPC) to evaluate temporal trends. The study is well-suited to inform health policy and cancer prevention strategies. However, the manuscript requires substantial revisions to improve clarity, scientific rigor, and structure. Issues related to writing quality, data interpretation, and discussion of health disparities must be addressed before the paper is suitable for publication.

Specific Comments

**1. Title and Abstract**

•The abstract is dense with data and could overwhelm readers. Please prioritize key findings and move granular statistics to the results section.

**Answer:** Thank you for your valuable suggestion. We have prioritized key findings by reorganizing the Results section: First, we presented the incidence and mortality trends across different racial groups, categorized by the six female-specific cancers. Second, we presented the deaths attributable to key risk factors trends from 2000 to 2021, categorized by the six female-specific cancers, lines 39-49.

•Clarify the study’s objective explicitly in the abstract.

**Answer:** We sincerely appreciate the reviewer's suggestion to clarify the study objective. As recommended: Added an explicit objective statement in the opening sentence of the Abstract: " This study aimed to analyze trends in incidence and mortality rates of six female-specific cancers (breast, cervical, uterine, ovarian, vaginal, and vulvar cancers) among diverse racial and ethnic groups in the United States, and to evaluate the attributable contributions of major risk factors to the cancer death burden as well as their temporal changes", lines 28-33.

•Language issues, such as “impacts on healthcare and economy,” should be revised for clarity (e.g., "healthcare systems and economic burden").

**Answer:** Thank you for your valuable feedback. We agree that the original phrasing "impacts on healthcare and economy" lacked clarity. As suggested, we have revised the sentence to: " Female-specific cancers (FSCs) impose substantial burdens on healthcare systems and economies worldwide.", line 25.

2. Introduction

•The background is informative but includes some redundancy. Streamline citations and focus on how this study fills gaps in existing literature. Also, use more current references.

**Answer:** Thank you for your constructive feedback. We have streamlined the citations by removing redundant references, and integrating more recent and relevant literature where appropriate. We have also sharpened the focus on the research gaps—particularly the lack of comprehensive, multi-cancer analyses and updated temporal trends across racial/ethnic groups—to better highlight the contribution of our study, lines 59-69, 78-80, references (4, 5).

•Define “disadvantaged populations” and consider referencing social determinants of health more explicitly to ground the disparities framework.

**Answer:** Thank you for your thoughtful comment. We have now defined the term “disadvantaged populations” in the context of social determinants of health （SDOH）, lines 70-80.

3. Methods

•Clarify statistical methods such as the Tiwari method and Joinpoint regression in plain terms for non-specialist readers.

**Answer:** Thank you for your suggestion. We have clarified methods (including the Tiwari method and Joinpoint regression) in the summary section, lines 127-130 and 136-139.

•Ensure that all data sources and coding schemes (ICD-10, ICD-O-3) are clearly defined.

**Answer:** Thank you for this valuable suggestion. We have revised the Methods section to provide clearer definitions of the data sources and coding schemes, as detailed below:

First, incidence data were obtained from the SEER 22-registry database, while mortality data were sourced from the SEER mortality database (reported by the NCHS), and the cancer burden attributable to the risk factors of four FSCs were from the GBD 2021, lines 89-92, 97-99 and 102-103.

Second, we have added clear definitions for both the ICD-O-3 and ICD-10 coding systems, stating their standard applications in cancer registries and mortality databases, respectively, lines 92-96, 99-101,105 and references (7, 8, 10).

In conclusion, the codes across all databases are not exactly the same, but they refer to the same cancer sites. SEER uses ICD-O-3 codes for cancer incidence, which are more specific to oncology and histology, whereas NCHS and GBD use ICD-10 codes for mortality data, which are more commonly applied in death reporting and health statistics.

•The rationale for examining both long and short-term trends should be better justified.

**Answer:** Thank you for your insightful comments. We have now added a clearer rationale in the manuscript explaining that: “To distinguish between enduring patterns and recent fluctuations, we analyzed both long-term and short-term trends.”, lines 121-122.

4. Results

•The results section is overly technical and could benefit from subheadings and clearer narrative transitions (e.g., "Incidence by Race", "Mortality by State").

**Answer:** Thanks for this excellent suggestion. We agree that more descriptive subheadings would greatly improve the narrative flow and readability of the Results section. The revised subheadings are as follows:

- 3.1 Incidence and Mortality by Race and Ethnicity, line 158.
- 3.2 Temporal Trends in Incidence and Mortality, line 192.
- 3.3 Incidence and Mortality by State, line 238.
- 3.4 Risk-Attributable Deaths Over Time, line 273.
- 3.5 Risk-Attributable Deaths by State, line 290.

•Ensure all acronyms (e.g., AAPI, AIAN, AAPC) are defined at first use in each section.

**Answer:** Thank you for pointing this out. We have now carefully reviewed the manuscript and ensured that all acronyms (including White, Black, AAPI, AIAN, AAPC, FSC etc.) are explicitly defined at their first use in each section of the manuscript. (e.g., Abstract, Introduction, Methods, Results, Discussion, Conclusion).

•Avoid inconsistent reporting formats (e.g., confidence intervals vs. brackets vs. parentheses).

**Answer:** Thanks for your meticulous comment. We have now conducted a thorough review of the entire manuscript and have standardized the reporting format for confidence intervals and other statistical values. Specifically, we have adopted the consistent format throughout the text, (e.g., (0.7; 95% CI, 0.6-0.7)). All instances using brackets or inconsistent parentheses have been corrected.

•Include visuals, such as maps or tables, referenced in the narrative for improved readability.

**Answer:** Thank you for this helpful suggestion. Although these figures were originally created and cited in the text, we have now optimized their placement by physically inserting them directly into the main body of the Results section as per the journal's format. This includes:

- The incidence and mortality trend figures in section 3.2.
- The U.S. state-level maps in section 3.3.
- The risk factor trend figure in section 3.4.
- The state-specific bar chart in section 3.5.

5. Discussion

•Expand the discussion on the root causes of disparities, including systemic inequities, access to screening and treatment, and socioeconomic factors.

**Answer:** Thank you for your insightful suggestion, which has greatly enriched our manuscript. We have substantially expanded our discussion on the root causes of disparities by explicitly integrating the concepts of structural barriers and systemic inequities throughout the section. The specific modifications include:

First, in the breast cancer paragraph, we refined the language to emphasize systemic factors: " Our findings confirm that breast cancer incidence remains highest among White women, while Black women experience disproportionately higher mortality—a pattern consistent with previous study. These disparities may be attributed to multiple factors, including socioeconomic status, cultural beliefs, and social inequities. Women with lower socioeconomic status face barriers to screening rates, later-stage diagnosis, and disparate treatments quality, contributing to poorer outcomes. Compared to White women, Black women are more likely to delay seeking immediate treatment for breast symptoms and experience higher rates of aggressive subtypes like triple-negative breast cancer. Regional variations in risk factors, such as alcohol consumption, may also contribute to geographic disparities. Rising breast cancer incidence is associated with increasing obesity, physical inactivity, and alcohol use. Notably, while deaths from all breast cancer risk factors declined from 2000–2021, potentially reflecting improved screening and therapies, the proportional contribution of metabolic and behavioral risks increased", lines 320-333.

Second, in the cervical cancer paragraph, we significantly strengthened the explanation for AIAN disparities: "Cervical cancer incidence decreased among most racial groups but increased among non-Hispanic American Indian and Alaska Native (AIAN) women, who also experience lower screening rates due to structural barriers such as geographic isolation, under-resourced healthcare, and socioeconomic challenges", lines 334-337. The addition of "structural barriers", "geographic isolation", and "under-resourced healthcare" provides a much more concrete and systemic explanation.

Last, in the vaginal cancer paragraph, we re-framed the discussion around structural access issues: "Persistently higher rates among Black and Hispanic women may stem from delayed diagnosis due to structural barriers to healthcare access, such as insurance coverage and regular care, leading to progression to invasive disease", lines 372-375. This change introduces the key term "structural barriers to healthcare access", moving the argument from individual-level access to system-level failure.

•Avoid overstatements or causal implications. For instance, “urgent need” can be

softened to “highlight the importance of...”

**Answer:** We thank the reviewer for this crucial guidance on academic language. We have meticulously reviewed the entire Discussion to ensure a cautious and objective tone by implementing the following specific changes:

First, we replaced imperative or definitive phrasing with more measured suggestions. For example, we now state that "State-level Medicaid expansion policies represent a potential lever for intervention" instead of using a stronger prescriptive phrase, line 355-356.

Second, we inserted words like "potential" (e.g., "potential lever for intervention") and "may" (e.g., "may stem from delayed diagnosis") to soften assertions and present findings as associations rather than definitive conclusions, lines 356, 373.

Last, in the vulvar cancer paragraph, we concluded with a neutral and objective statement that "the exact reasons for these divergent trends require further study", openly acknowledging the limits of our current knowledge and avoiding overinterpretation, lines 386-387.

•Acknowledge the strengths of the study (e.g., large datasets, national scope).

**Answer:** We are grateful for this positive feedback. As per the suggestion, we have now explicitly acknowledged the core strengths of our study by adding the following sentence to the very first paragraph of the Discussion: "This study utilized high-quality, nationally representative data spanning over two decades, enabling a comprehensive analysis of temporal trends in incidence and mortality for six major female-specific cancers (FSCs) in the US", lines 313-315.

6. Conclusion

•Strengthen the conclusion by offering specific recommendations or implications for policy, practice, or future research (e.g., expand HPV vaccination, improve Medicaid coverage).

**Answer:** Thank you for this excellent suggestion to enhance the practical impact of our conclusion. In direct response, we have replaced generic statements with highly specific, actionable recommendations:

First, we now explicitly recommend "expanding HPV vaccination in underserved communities" to address disparities in HPV-associated cancers, "implementing Medicaid coverage expansion to improve treatment access" to overcome financial barriers to care, and "developing targeted obesity prevention initiatives in high-risk regions" to counter a major shared risk factor, lines 400-402.

Second, we have defined clear priorities for investigation, specifically proposing studies to examine "structural barriers in AIAN populations," assess "state-level policy impacts on uterine cancer," and investigate "vaginal/vulvar cancer risk factors in vulnerable groups", lines 403-405.

•Emphasize the importance of equity-driven cancer control efforts.

**Answer:** We appreciate the reviewer's guidance to sharpen our focus on equity. We have thoroughly revised the conclusion to frame all recommendations within an equity-driven context. The concept of targeting vulnerable populations is now the central theme:

First, we explicitly state that our recommended interventions (e.g., vaccination, Medicaid expansion) are targeted toward "underserved communities" and "vulnerable groups", lines 400-405.

Second, we emphasize that reducing disparities requires strategies designed to overcome structural barriers, namely through "culturally tailored navigation programs and community-based screening", line 406.

Finally, we have defined the ultimate goal of comprehensive efforts as achieving "progress toward equity goals," moving beyond general cancer control to explicitly aim for equitable outcomes for all populations, line 409.

7. Language and Style

•The manuscript would benefit from professional English editing to correct awkward

phrasing, verb tense inconsistencies, and repetitive language.

**Answer:** We thank the reviewer for this helpful feedback regarding the language and style of our manuscript. We have carefully addressed these concerns through a thorough, professional revision of the text. Specifically, we have:

- Corrected awkward or unclear phrasing to improve readability and fluency.
- Ensured consistency in verb tenses throughout the manuscript.
- Eliminated repetitive language by varying word choice and refining sentence structures.

In addition to these targeted improvements, the manuscript has been polished overall to enhance clarity, tone, and academic style. We believe the revised version now meets the high standards of language quality required for publication.

•Suggested revision examples:

“Raised among AAPIs” → “Increased among Asian American and Pacific Islander

populations”

“Had higher burdens” → “Experienced higher incidence and mortality rates”

**Answer:** We sincerely thank the reviewer for these insightful and constructive suggestions for improving the clarity and precision of our language. We have carefully reviewed the entire manuscript and applied the principles demonstrated in your examples:

1. We have replaced informal or vague terms with more formal and precise academic language.
2. We have now ensured that all acronyms (e.g., AAPI) are explicitly defined at their first use in each section of the manuscript for clarity.

The specific examples you provided were particularly helpful, and we have extended this level of scrutiny to the entire text to enhance its overall readability and professionalism.

Minor Issues

•Ensure that all figures and tables referenced (e.g., Table 4, Figures 1–4) are included and clearly labeled.

**Answer:** We thank the reviewer for their meticulous attention to detail. We have double-checked the entire manuscript and confirm that all figures and tables (Table 1-4, Figures 1-6) are now: Included in the correct order within the manuscript file. Clearly labeled with descriptive titles and legends that fully explain the presented data. Referenced accurately in the main text, ensuring that every mention in the narrative corresponds to the correct visual element.

•Abbreviations should be consistent throughout (e.g., “FSCs” vs. “female-specific cancers”).

**Answer:** We appreciate this suggestion for improving consistency. We have now standardized the use of all acronyms throughout the manuscript. The full term, such as“female-specific cancers,” is used upon first appearance in each major section (Abstract, Introduction, Methods, etc.), and the abbreviated form “FSCs” is used consistently thereafter to improve readability and avoid repetition.

•Clarify any suppressed data points and include a rationale (e.g., <16 cases suppressed per SEER guidelines).

**Answer:** We thank the reviewer for highlighting this important requirement for data reporting. We have now added a clarification in the Methods section that states:
“When the numbers of cases or deaths used to compute rates are small, those rates tend to have poor reliability. Therefore, to discourage misinterpretation or misuse of rates or counts that are unstable, incidence and death rates and counts are not shown in tables and figures when the case or death counts are below 16”, lines 138-142.

Thanks again for your kindest comments and constructive suggestions to improve the quality of this study.

**Replies to Reviewer #2**

I've reviewed your manuscript, and I'm happy to report that it looks great!

Here are some specific things that caught my attention:

- Your manuscript is technically sound, and the data presented support your

conclusions. You've done a great job using high-quality population-based incidence

and mortality data from reputable sources like SEER and GBD to investigate disparities

and trends in female-specific cancers.

- Your study is a solid piece of scientific research that uses established databases and

statistical analysis methods to get the job done. I appreciate that you've used Joinpoint

Trend Analysis Software and the Tiwari method to estimate temporal trends and

calculate rate ratios.

- Your statistical analysis looks rigorous and well-done. You've accounted for potential

confounders and used standard epidemiological methods, which is great to see.

- Since you're using publicly available data from SEER and GBD, it's easy for readers to

access the data underlying your findings. Kudos for transparency!

As for suggestions, I've got a few minor ones to help make your manuscript even

stronger:

- Some of your sentences are a bit long and could be broken up for better readability.

Consider taking a look at those to see where you can make some tweaks.

**Answer:** We thank the reviewer for this excellent suggestion. We have thoroughly reviewed the manuscript and identified several long or complex sentences, particularly in the Introduction and Discussion sections. These sentences have been broken down into shorter, clearer statements to significantly improve the flow and readability of the text. (Example: We have revised sentences in paragraphs [Page 2, line 25-27; Page 4, lines 62-66; Page 5, lines 81-83; Page 6, lines 114-115 etc.] to enhance clarity).

- Double-check your formatting and style throughout the manuscript to ensure

consistency. It's a small thing, but it makes a big difference in the overall presentation.

**Answer:** We appreciate the reviewer's attention to detail. We have performed a comprehensive check of the entire manuscript to ensure strict consistency in formatting and style. This includes verifying uniformity in: Heading levels (font size and bolding); reference formatting; figure and table captions; abbreviations and terminology. We confirm that the manuscript now adheres to a consistent style throughout.

- Your conclusion does a great job summarizing your main findings, but consider

rephrasing some sentences to make them even clearer.

**Answer:** We are grateful for this helpful comment. We have revisited the conclusion section and rephrased key sentences to enhance their clarity and impact, ensuring our main findings and their implications are communicated as effectively as possible. The revised conclusion are as follows:

“Using high-quality population-based data, our study revealed a strong heterogeneity in incidence/mortality trends for six female-specific cancers (FSCs) across the US. Further efforts to prevent FSCs should focus on the management of risk factors and intervention measures, including expanding HPV vaccination in underserved communities, implementing Medicaid coverage expansion to improve treatment access, and developing targeted obesity prevention initiatives in high-risk regions. Further research should elucidate these patterns and mitigate disparities, particularly studies examining structural barriers in AIAN populations, state-level policy impacts on uterine cancer, and vaginal/vulvar cancer risk factors in vulnerable groups. Meanwhile, culturally tailored navigation programs and community-based screening can reduce racial/ethnic mortality disparities through improved care access. A comprehensive long-term approach requires obesity prevention and continuous registry monitoring to evaluate progress toward equity goals.”

Once again, we thank for your time and valuable comments, which have undoubtedly strengthened our manuscript.

Reference

4.	Korn AR, Walsh-Bailey C, Correa-Mendez M, DelNero P, Pilar M, Sandler B, et al. Social determinants of health and US cancer screening interventions: A systematic review. CA Cancer J Clin. 2023;73(5):461-79.

5.	Ellington TD, Henley SJ, Wilson RJ, Miller JW, Wu M, Richardson LC. Trends in breast cancer mortality by race/ethnicity, age, and US census region, United States─1999-2020. Cancer. 2023;129(1):32-8.

7.	Huang Y, Huang H, Wei T, Zhang A, Zhang H, Zhang Z, et al. Disparities, Trends, and Predictions to 2040 in Gastrointestinal Cancer Incidence, Mortality in the United States. The American journal of gastroenterology. 2024;120(6):1367-80.

8.	Jemal A, Ward EM, Johnson CJ, Cronin KA, Ma J, Ryerson B, et al. Annual Report to the Nation on the Status of Cancer, 1975-2014, Featuring Survival. J Natl Cancer Inst. 2017;109(9).

10.	Sun P, Yu C, Yin L, Chen Y, Sun Z, Zhang T, et al. Global, regional, and national burden of female cancers in women of child-bearing age, 1990-2021: analysis of data from the global burden of disease study 2021. EClinicalMedicine. 2024;74:102713.
